# Interferon and Biologic Signatures in Dermatomyositis Skin: Specificity and Heterogeneity across Diseases

**DOI:** 10.1371/journal.pone.0029161

**Published:** 2012-01-03

**Authors:** David Wong, Bory Kea, Rob Pesich, Brandon W. Higgs, Wei Zhu, Patrick Brown, Yihong Yao, David Fiorentino

**Affiliations:** 1 Department of Dermatology, Stanford University School of Medicine, Stanford, California, United States of America; 2 Department of Biochemistry, Stanford University School of Medicine, Stanford, California, United States of America; 3 MedImmune, Translational Sciences, One MedImmune Way, Gaithersburg, Maryland, United States of America; University of Lyon, France

## Abstract

**Background:**

Dermatomyositis (DM) is an autoimmune disease that mainly affects the skin, muscle, and lung. The pathogenesis of skin inflammation in DM is not well understood.

**Methodology and Findings:**

We analyzed genome-wide expression data in DM skin and compared them to those from healthy controls. We observed a robust upregulation of interferon (IFN)-inducible genes in DM skin, as well as several other gene modules pertaining to inflammation, complement activation, and epidermal activation and differentiation. The interferon (IFN)-inducible genes within the DM signature were present not only in DM and lupus, but also cutaneous herpes simplex-2 infection and to a lesser degree, psoriasis. This IFN signature was absent or weakly present in atopic dermatitis, allergic contact dermatitis, acne vulgaris, systemic sclerosis, and localized scleroderma/morphea. We observed that the IFN signature in DM skin appears to be more closely related to type I than type II IFN based on in vitro IFN stimulation expression signatures. However, quantitation of IFN mRNAs in DM skin shows that the majority of known type I IFNs, as well as IFN g, are overexpressed in DM skin. In addition, both IFN-beta and IFN-gamma (but not other type I IFN) transcript levels were highly correlated with the degree of the *in vivo* IFN transcriptional response in DM skin.

**Conclusions and Significance:**

As in the blood and muscle, DM skin is characterized by an overwhelming presence of an IFN signature, although it is difficult to conclusively define this response as type I or type II. Understanding the significance of the IFN signature in this wide array of inflammatory diseases will be furthered by identification of the nature of the cells that both produce and respond to IFN, as well as which IFN subtype is biologically active in each diseased tissue.

## Introduction

Dermatomyositis (DM) is a chronic inflammatory disorder that can affect the skin, muscle, and other organs and is associated with significant morbidity and mortality. The prevalence of DM is not well-defined, as it is historically grouped together with polymyositis (PM) and inclusion body myositis (IBM) in most epidemiologic studies. The estimated incidence of DM is approximately 1 per 100,000 per year [1]. There are two forms of DM, juvenile and adult, that have overlapping but some distinct clinical features [1]. DM is considered an autoimmune disease, as it is associated with specific autoantibodies, and its prevalence is associated with particular HLA alleles [2].

Currently classified as an idiopathic inflammatory myopathy (IIM), much of the work in understanding DM has been focused on the muscle pathology that accompanies this disorder. Some authors have suggested that the muscle disease is due to an immune-mediated vasculopathy, with resultant ischemic damage to the muscle fibers resulting in myocyte death and muscle atrophy [3]. However, the precise mechanism of either endothelial cell or myocyte damage is unclear [3]. Inflamed muscle shows infiltration with B lymphocytes, T lymphocytes, and dendritic cells, and the contribution of each to the disease is not well understood [4], [5]. Cytokines and chemokines are also postulated to be important in disease pathogenesis [6]. DM muscle expresses large amounts of type I interferon (IFN)-inducible genes [7]. It is possible that these gene products might themselves be causing the vascular and parenchymal cellular damage [7]. In addition, an IFN signature that correlates with overall disease activity is observed in peripheral blood of most DM patients, including patients with juvenile DM [8], [9], [10]. Thus, much as with other autoimmune diseases such as Systemic Lupus Erythematosus and Sjogren's syndrome, DM is emerging as a potentially type I IFN-driven autoimmune disease.

The pathogenesis of skin inflammation in DM is not well-studied and its pathologic mechanisms may or may not overlap with those causing DM muscle disease. The typical cutaneous histopathologic changes in DM include pathologic apoptosis/necrosis of keratinocytes, perivascular and lichenoid inflammation, increased dermal mucin deposition, endothelial cell damage with loss of capillaries, and vascular dilatation [11], [12]. Similarities to muscle disease exist in the skin: first, vasculopathy and vascular deposition of complement components can be detected in cutaneous DM skin [13], [14]; second, there is damage to the parenchymal cells (e.g. keratinocytes) [11]; third, DM skin appears to be characterized by increased abundance of several gene products that are known to be upregulated by IFN [15], [16] as well as by increased numbers of plasmacytoid dendritic cells [17], [18]. It has been proposed that some of these gene products, such as CXCL9/10/11, act as chemoattractrants for CXCR3-bearing T lymphocytes which can then perpetuate inflammation and keratinocyte necrosis [16]. In addition, there appears to be a topographical relationship between the site of cell injury, inflammation, and the basement membrane that is shared by both skin and muscle disease in DM [19]. However, there are certain differences between muscle and skin disease in DM patients: clinically, the course of skin and muscle disease is often discordant between patients [20], suggesting different mechanisms of disease pathogenesis; in addition, there are important histopathologic differences between skin and muscle—for example, B lymphocytes are rarely found in the skin disease [21], in contrast to their common perivascular location in DM muscle. Current therapies for DM skin disease include immunosuppressive medications, which are not uniformly efficacious and can be associated with significant morbidity [20]. A better understanding of molecular pathogenesis of DM could potentially unveil better molecular markers for this disease and more effective targets for therapy.

In this study, we performed global gene expression analysis of skin from DM patients and healthy controls. Our results reveal a gene expression signature that is unique to skin inflammation in both DM and lupus, but distinct from other inflammatory skin diseases. Within this signature, we also identify a characteristic IFN-driven expression pattern in DM and lupus that partially overlaps with the pattern seen in other lichenoid dermatoses as well as psoriasis.

## Results

### Defining expression signature in skin of individuals with DM

**Figure 1:**
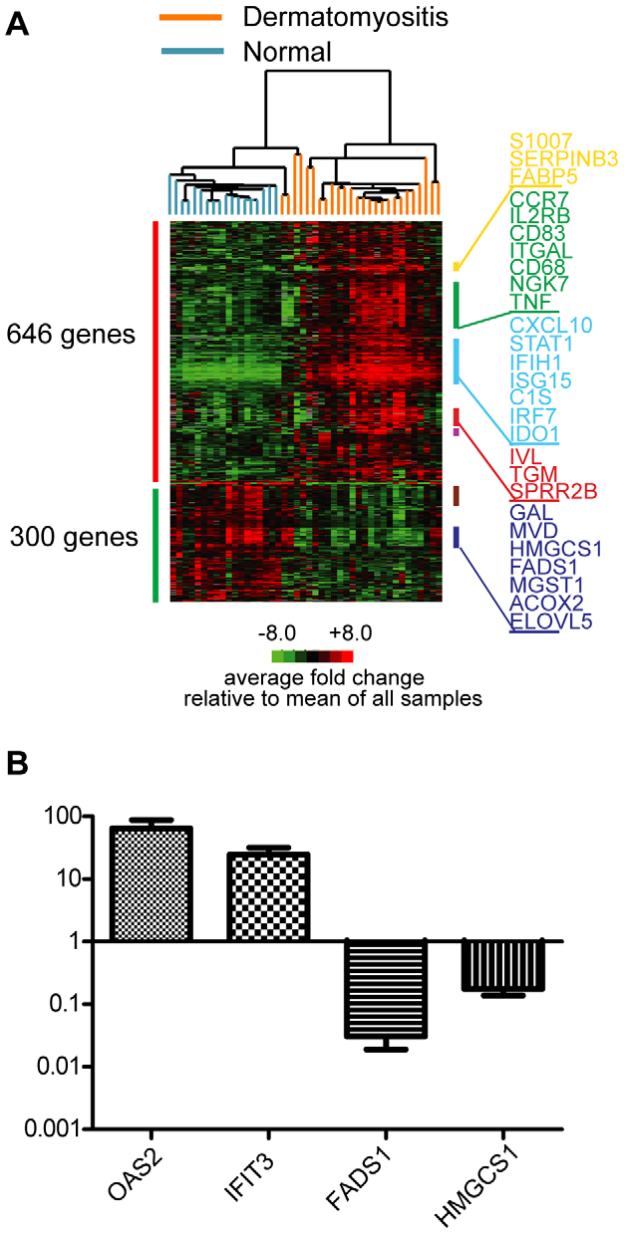
Visualization and validation of DM gene expression on HEEBO oligonucleotide arrays. RNA was prepared from skin biopsies as detailed in Methods, and the same source was used for both gene expression array (**A**) and QRT-PCR experiments (**B**). **A.** Experimental hierarchical clustering dendrogram. Two-dimensional hierarchical clustering was performed on gene expression data from active DM skin lesions and skin from healthy controls. A set of 946 genes whose average expression significantly differed between DM and healthy controls (the “DM module) was used to group sample expression data; 646 genes were upregulated (red bar on left) and 300 genes were downregulated (green bar on left) in DM patients relative to control biopsies. All values are in log_2_ space and are mean-centered across each gene. Colored bars on right indicate gene clusters evident on dendrogram: yellow bar—epidermal activation; green bar—leukocyte function; light blue bar—IFN signature; red bar—epidermal differentiation; lavender bar—immunoglobulin; brown bar—ribosome; dark purple bar-lipid metabolism. A larger view of the dendrogram and more complete lists of genes in these clusters can be found in **Supplementary Figure S2**. **B.** Validation of array data using TaqMan QRT-PCR of selected transcripts. QRT-PCR was performed (see Methods) on 9 DM skin RNA samples and 8 control skin RNA samples for four selected transcripts that were either found to be upregulated (OAS2, IFIT3) or downregulated (FADS1, HMGCS1) in DM skin. Shown are the mean values (with SEM) for each transcript in DM skin relative to the mean value in control skin. Relative transcript values for each of the 4 genes across the 9 DM samples showed a high correlation (Pearson's r = 0.71 to 0.86) between the HEEBO array and QRT-PCR.

In order to understand the molecular characteristics of DM, we hybridized RNA from skin biopsies from patients with active DM (n = 16) and healthy patients (n = 10) (total of 32 independent biopsies, with 12 technical replicates for total of 44 arrays) to HEEBO oligonucleotide arrays. The baseline characteristics of these patients are listed in Table S1. Using significance analysis of microarrays (FDR<0.05), we identified 946 unique genes that were differentially regulated at least 2-fold in DM skin relative to healthy skin: we term this the “DM gene module” (Table S2). Over two-thirds (646 of 946) of the genes identified were up-regulated and 300 were down-regulated in DM skin relative to healthy skin. Two-dimensional hierarchical clustering using these 946 genes segregated all healthy controls and DM patients into 2 distinct clusters (Fig. 1A). The 3 DM patients (4 samples) that segregated with the healthy controls formed a separate sub-cluster unto themselves. There was no apparent clustering with regards to age, gender, or site of biopsy (not shown). When patients with inactive skin disease were included in the cluster analysis, we found that 6 of 7 of the inactive DM patients clustered with the healthy controls (Fig. S1).

We noted that the DM gene module contained groups of genes that are known to function in several distinct biological pathways (Figs. 1A and S2). For example, a group of 13 genes associated with keratinocyte/epidermal activation (S100A7/8/9, SERPINB3/4, and FABP5) was upregulated in DM (yellow bar, Figs. 1A and S2). In addition, genes involved in epidermal differentiation, including IVL, TGM, SPRR2A/B/E/G, SERPINB7/8, are induced in DM (red bar, Figs. 1A and S2), which have also been shown to be elevated in other inflammatory skin diseases, such as psoriasis and atopic dermatitis [22], [23]. A large cluster of genes upregulated in DM are involved in T cell (IL2RB, CD2, TRAC, CD3D, CTLA4), cytotoxic/NK cell (NKG7), macrophage (CD68), and dendritic cell (CD83) function (green bar, Figs. 1A and Fig. S2), which is consistent with previous observations regarding T cell and macrophage infiltration in DM skin [21]. The overexpression of the cell surface marker CD83 is consistent with previous studies that showed with immunostaining that mature dendritic cells are present in DM skin (either myeloid or plasmacytoid) [16], [18]. In addition, skin from patients with DM had induction of markers of endothelial cell activation (VCAM1, SEL-L). A subset of the DM samples showed elevated immunoglobulin expression, indicative of infiltrating mature B lymphocytes and/or plasma cells, the former of which have been shown to be present in low and variable amounts in DM skin (lavender bar, Figs. 1A and S2) [21].

A striking observation in DM skin was the over expression of genes that were induced by IFN (light blue bar, Figs. 1A and S2). In fact, 21 of the top 25 most upregulated genes are known or presumed to be upregulated by IFN [24], [25], [26] (Table S3). These genes included CXCL10, IFIH1, ISG15/UBE2L6, C1S/R, IRF7, IDO1, MXB and CHN1. Notably this cluster of IFN genes was generally not overexpressed in the skin of DM patients with inactive skin disease (Fig. S1), suggesting that they are induced preferentially in active skin lesions.

Of the downregulated genes in DM, many were involved in either ribosomal synthesis (brown bar, Figs. 1A and S2) or lipid metabolism (dark purple bar, Figs. 1A and S2). The latter group of genes included MVD, MGST1, GCS1, FADS1/2 and GAL. Many of the genes involved in lipid metabolism have been previously reported to be downregulated in other inflammatory skin disorders such as psoriasis and atopic dermatitis [22], [27].

To validate our findings from these microarrays, we performed TaqMan quantitative real-time reverse-transcriptase PCR-(QRT-PCR-) based assays on several genes from two of the enriched gene modules, the IFN and lipid gene modules (Fig. 1B). As suggested by the arrays, TaqMan QRT-PCR confirmed that two IFN-induced genes (IFIT3 and OAS2) were upregulated, and two genes involved in lipid metabolism (FADS, HMGCS1) were downregulated in the skin of DM patients. Across patient samples, there was a good correlation between microarray and QRT-PCR values for each of the four genes (Spearman ρ = 0.76 to 0.88).

**Figure 2:**
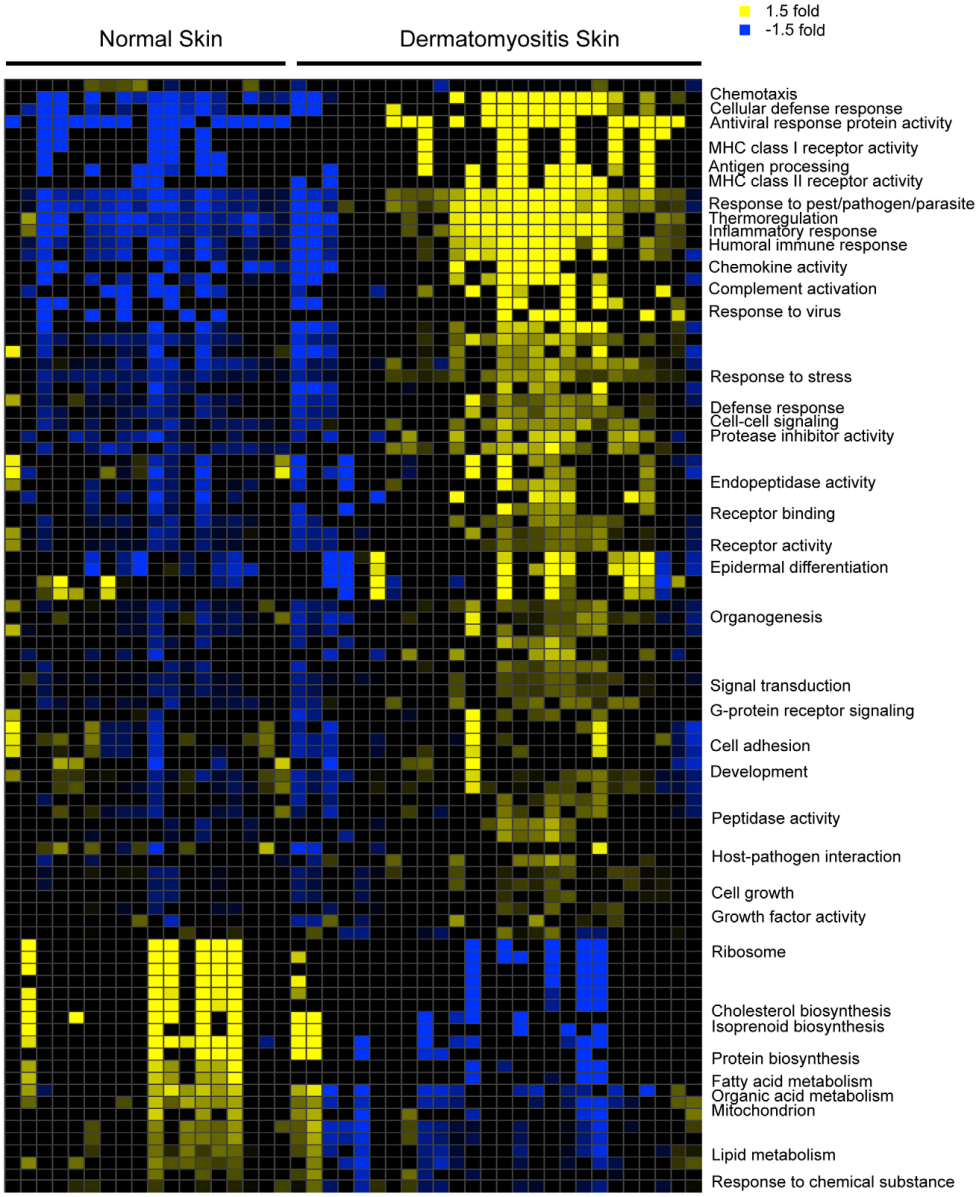
DM skin module map. Module map of the Gene Ontology (GO) and Kyoto Encyclopedia of Genes and Genomes (KEGG) Biological Processes differentially expressed among the active DM samples is shown. Each column represents a single microarray (e.g. patient) and each row represents a single GO or KEGG biological process. Only modules that were significantly enriched (minimum 2-fold change, p = 0.05) on at least 4 microarrays are shown. The average expression of the gene hits from each enriched gene set is displayed here. Only gene sets that show significant differences after multiple hypothesis testing were included. Selected GO or KEGG biological processes are shown. The entire figure with all biological processes can be viewed in Supplementary Figure S3.

In order to systematically investigate the biological processes that are altered in DM skin, we constructed a gene module map (p<0.05, FDR<0.05) to look for enrichment of gene sets associated with various biologic pathways and/or functions, based on either Gene Ontology (GO) and KEGG pathway annotations and on data derived from publicly available gene expression studies (Figs. 2 and S3). Biologic functions and pathways found to be enriched in the upregulated genes in DM skin included antigen processing, complement activation, MHC I/II receptor activity, T cell function, chemokine activity, antiviral and immune responses and epidermal differentiation (Fig. 2). Besides the IFN signaling pathway, TNF-alpha, IL-1, IL6, IL17 and VEGF pathway activation was also observed in the skin of DM patients (Fig. S3). Downregulated processes in DM skin included fatty acid metabolism, lipid biosynthesis and metabolism, mitochondrial function (electron transport and pyruvate metabolism), peroxisome and ribosomal function.

### Mapping the DM gene module across different skin diseases

**Figure 3:**
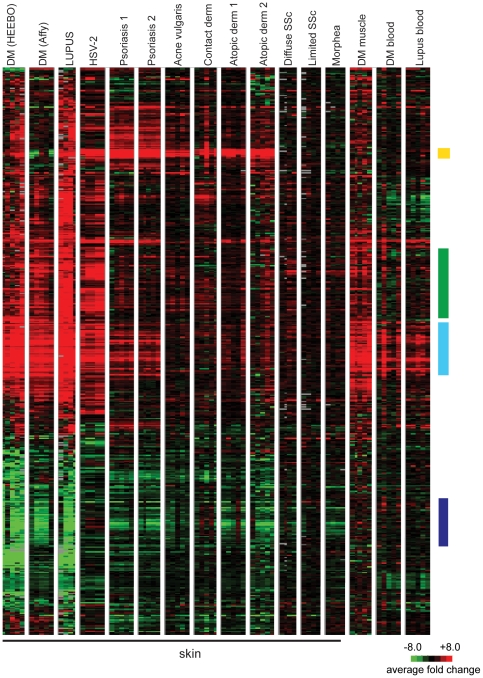
Mapping the DM module across different inflammatory disease tissues. Shown is a hierarchical clustering dendrogram of gene expression data from DM and multiple other diseases. The genes visualized represent all of the genes of the DM module that are common to all of the array platforms used to generate the data shown. The expression pattern of this set of 490 genes across all of the disease states shown was clustered using average linkage clustering, while the columns (samples) were not clustered and grouped by disease and experimental dataset. Expression data for each gene is relative to the mean expression level for all healthy controls (red = upregulated; green = downregulated) within each dataset, with the exception that HSV-2 data is relative to uninvolved skin of *diseased* HSV-2 patients (see Methods). Each disease state is composed of five columns, representing five representative examples (patients) of each disease. DM (HEEBO) and DM (Affy) represent data from independent DM skin biopsies run on either HEEBO or Affymetrix arrays, respectively. The remaining datasets were obtained from publicly available GEO omnibus data (see Methods). All data are derived from skin biopsies with the exception of the three diseases on the right, as indicated. Colored bars on right indicate gene clusters of the DM module that were discussed in the text for Figure 1: yellow bar—epidermal barrier; green bar—leukocyte function; light blue bar—IFN signature; dark purple bar-lipid metabolism.

We next sought to study the specificity of these gene expression changes across various inflammatory skin diseases. With the exception of the cutaneous lupus samples (which were internally collected and processed along with the DM samples), we used publicly available gene expression sets, and all disease data were normalized to internal healthy controls within each study. We examined the expression of genes in the DM gene module across the different diseases (Fig. 3; see also Fig. S4 for a full pairwise correlation matrix). Due to the use of different array platforms in this analysis, only 490 genes of the original 946 genes of the DM module were contained in all of the experimental datasets analyzed and could thus be used for the analysis. Although the data were generated by different investigators on different microarray platforms, diseases for which more than one dataset existed (psoriasis and atopic dermatitis) showed remarkably similar expression patterns between datasets (Spearman ρ = 0.77 to 0.82). A similar correlation analysis between DM samples analyzed using HEEBO arrays and an independent set of DM samples analyzed using Affymetrix arrays demonstrated high correlation (Spearman r = 0.76) (Fig. 3). This suggests that the differences observed between diseases were primarily driven by biological disease-specific characteristics rather than platform or laboratory differences.

The gene expression profiles in DM and lupus skin samples were remarkably similar (Spearman r = 0.87; Fig. 3), suggesting that skin from individuals with DM and lupus, which are both autoimmune interface dermatitis processes, have a common pathophysiology. Interestingly, the upregulated genes in the DM gene module were similarly induced in the muscle of DM patients (Spearman r = 0.56; Fig. 3). DM skin also showed similarity to samples from (herpes simplex-2) HSV-2 infection (Spearman r = 0.64; Fig. 3). The rest of the inflammatory skin diseases appeared to have a small subset of similarly regulated genes as in DM, but overall the pattern was quite different. The epidermal activation genes, such as S100 and SERPIN genes appear to be generally upregulated across most of the skin diseases except for localized and systemic scleroderma (yellow bar, Fig. 3). In addition, many of the inflammatory genes were variably expressed across different diseases, with the highest expression observed in cutaneous lupus and HSV-2, moderately high expression in DM skin and muscle, and modest increase in expression in other diseases (green bar, Fig. 3). Genes involved in the complement cascade (C1S, C1R, C1QB, and C2) were only upregulated in DM skin/muscle, cutaneous lupus, and HSV-2 skin (not shown). IFN-inducible genes were very highly overexpressed in DM skin/muscle, lupus skin, and HSV-2 skin; and to a lesser degree in psoriasis, and the blood of DM and lupus patients (light blue bar, Fig. 3). Genes of lipid metabolism were generally repressed not only in cutaneous DM and lupus but also psoriasis, atopic dermatitis, and morphea, and to a variable extent in other inflammatory skin diseases (dark purple bar, Fig. 3). Of note, this repression of lipid metabolism genes is not found in DM muscle, DM blood, or lupus blood, and may thus be a skin-specific phenomenon. Thus, a cross disease comparison reveals that some gene expression changes may be more commonly seen in inflammatory skin diseases, while other (e.g. IFN signature) are more specific, and overall, DM and lupus skin share a highly unique gene expression signature.

### Measuring the IFN signature across different skin diseases

**Figure 4:**
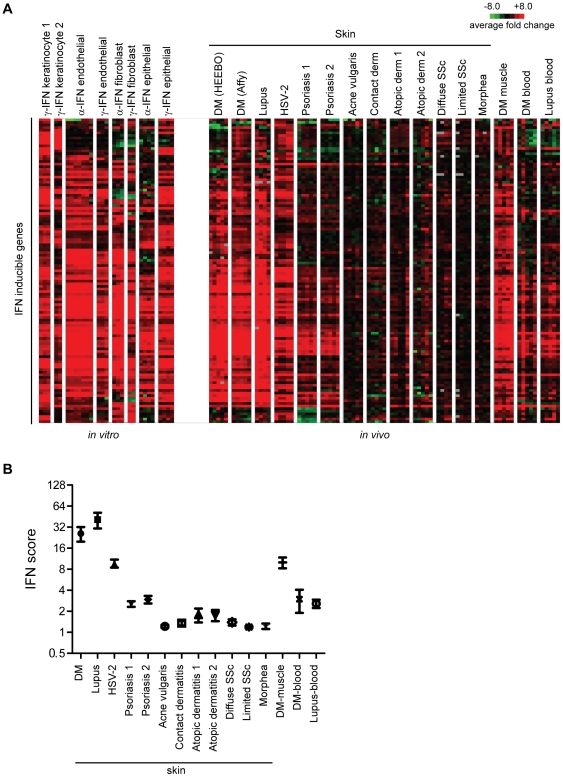
Visualizing the pattern and strength of the IFN signature expression across multiple disease states. **A.** Heatmaps of gene expression of the IFN inducible genes both *in vitro* and *in vivo* across diseases. The expression of a set of 117 genes comprising the “core IFN signature” (labeled as “IFN inducible genes” in the figure) is shown following one-way hierarchical clustering (genes only) using the entire *in vitro* and *in vivo* dataset. The left panel shows the expression patterns of this IFN signature following various *in vitro* stimulations with IFN-alpha or IFN-gamma on different responding cell types, as indicated. The right panel shows the expression patterns of the IFN signature across multiple disease states. The data from the disease states were derived from publicly available data as described in Figure 3 and Methods. **B.** Comparing the strength of the IFN signature across disease states using an IFN score. The top 25 expressed IFN-inducible genes (a static list) was derived from the same *in vitro* data used to generate the core IFN signature as described in Methods. The median expression value (in linear space) of this set of 25 genes within each disease sample was defined as the IFN score for that sample. Shown is the mean and SEM for IFN scores calculated for each disease dataset represented in panel A.

Although many IFN-inducible genes were upregulated in our DM module, we wished to determine in more detail how expression of a pre-defined module of IFN-inducible genes was regulated in DM and several other inflammatory skin diseases. A consensus list of 117 IFN-stimulated genes (Table S4) was derived by using publicly available data from in vitro stimulations of various cell types with either type I or II IFNs (see Methods). The expression pattern of these IFN-inducible genes was then surveyed across both the *in vitro* IFN stimulation data as well as diseased skin expression data. Some variation exists in the *in vitro* response upon IFN stimulation, depending on the cell type, IFN type, and the independent labs that generated the data (Fig. 4A, left panel). Again it appeared that DM skin and muscle, lupus skin, and HSV-2 skin infection had the most robust overexpression of IFN-inducible genes (Fig. 4B, right panel; also see Fig. S5 for a full pairwise correlation matrix). As was the case for the entire DM module, the expression patterns of IFN-inducible genes in skin of DM and lupus patients appeared almost indistinguishable, and similar to those in DM muscle and HSV-2. DM blood and lupus blood had a weaker induction of IFN genes, which is likely due to the fact that the IFN module was constructed using data from cell types present in skin and not blood. Interestingly, psoriasis demonstrated induction of a subset of IFN-inducible genes. In contrast, most of the skin samples of acne vulgaris, contact dermatitis, atopic dermatitis, allergic contact dermatitis, systemic sclerosis, and morphea did not appear to show significant induction of IFN-driven genes.

We next quantified the strength of the IFN-signature across different disease states. Similar to previous studies, we selected the top 25 most highly IFN-inducible genes from our “core IFN signature”, based on the *in vitro* studies that were used to define this signature (Table S4) [28]. We found that the scores calculated these 25 genes versus the full 117 gene module display a high correlation (Spearman's r = 0.96; not shown). We then calculated the median expression value (relative to healthy controls) of these 25 genes for each sample, which constituted the “IFN score” (Fig. 4B) [28]. DM and lupus skin had the highest average median IFN scores of 20 and 44, respectively. DM muscle and HSV-2 skin had high IFN scores (9.5 and 8.1, respectively), while the two psoriasis datasets yielded mean scores of 2.5 and 2.8. In contrast, all other skin diseases had scores of 1.7 or less.

**Figure 5:**
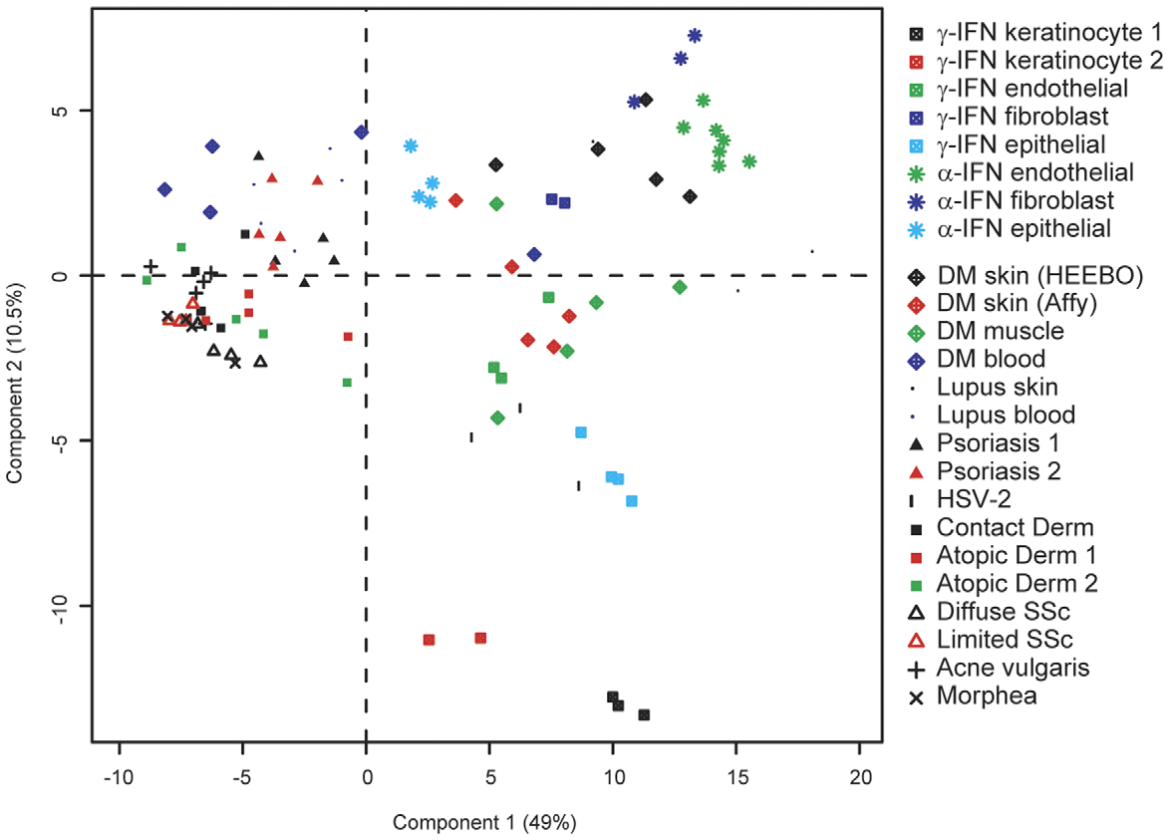
Distinguishing between type I and II IFN expression patterns across disease states. Two-dimensional PCA plot is shown using expression data derived from our data (DM, lupus) and publicly available datasets across the 117 gene “core IFN gene set” as described in Methods. Approximately 60% of the total data variation is summarized in these first two principal components.

In addition to the differences in overall IFN score, we were intrigued with the different patterns of IFN-induced genes in the various skin inflammatory diseases (Fig. 4A). These different patterns of IFN signature could be due to multiple factors, including: 1) a difference in cell types responding to IFN stimulation, 2) a difference in IFN subtypes (e.g. type I or II) that are present among the diseases, or 3) the presence of other cytokines that may induce a subset of genes in our “core IFN set”. To further investigate the differences in these patterns, we performed principal components analysis of the various disease samples as well as the samples obtained using in vitro IFN stimulations (Fig. 4A) using the *in vitro*-defined core IFN signature genes (Fig. 5A). We observed that the horizontal component tends to correlate with the strength of IFN signature, specifically in the skin (Figs. 4B and 5). Thus, DM muscle, DM and lupus skin, and HSV-2 are furthest to the right, while psoriasis, DM and lupus blood, and selected systemic sclerosis and atopic dermatitis samples all are modestly displaced to the right from all other skin diseases that are clustered together on the left—this is in general agreement with our IFN scoring data (Fig. 4B). On the other hand, the vertical component (component 2) appears to at least partially distinguish type I versus type II IFN (see *in vitro* stimulation data towards the right of Fig. 5). Additionally, the disease samples tend to be more positively positioned along this component, suggesting that DM, lupus and psoriasis all have signatures more consistent with type I IFN than with type II IFN.

### IFN gene expression in DM skin

**Figure 6:**
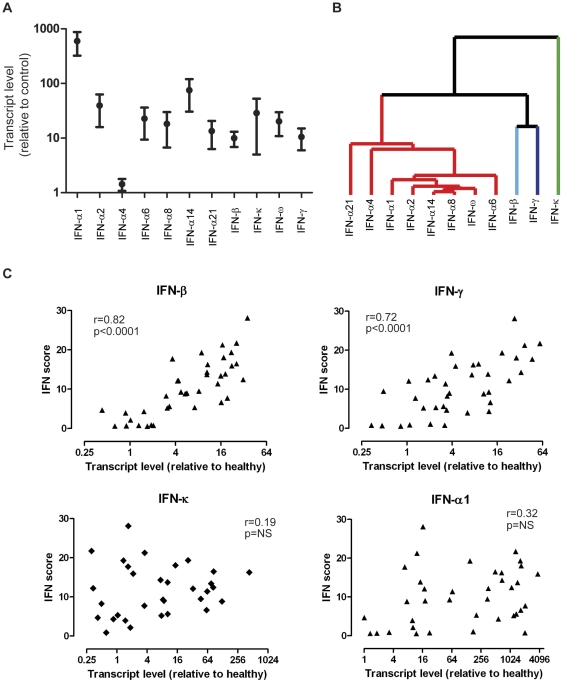
Expression of IFN transcripts in DM skin and correlation with downstream response. **A.** Upregulation of most IFN transcripts in DM skin. QRT-PCR analysis of different IFN transcripts in DM skin samples. TaqMan PCR was performed (see Methods) on 39 DM skin RNA samples and 4 control skin RNA samples for selected IFN transcripts. Shown are the mean values (with SEM) for each transcript in DM skin relative to the mean value in control skin. **B.** IFN transcripts are not co-regulated across DM skin samples. Hierarchical clustering dendrogram is shown for relative IFN transcript expression levels (using QRT-PCR) across 39 DM samples. Average linkage clustering was performed using correlation (uncentered) similarity metric. Similarity branches (length is inversely correlated with similarity of expression patterns) for IFN-α and IFN-ω are shown in red, IFN-beta in light blue, IFN-gamma in dark purple, and IFN-κ in green. **C.** IFN-beta and IFN-gammacorrelate most closely with the IFN score in DM skin. For each RNA sample made from 39 independent DM skin biopsies, an IFN score was calculated based on transcript levels of downstream IFN-inducible genes from array data (as described in Figure 4B and Methods) as well as a relative transcript level (using QRT-PCR) for several IFN transcripts (see panel A). Shown are correlation plots (for each of the 39 samples) of the IFN score and IFN transcript levels for IFN-a1, IFN-beta, IFN-gamma, and IFN-kappa.

In order to further characterize the IFN response in DM skin, we performed QRT-PCR analysis of the selected IFNs, including IFN-alpha (subtypes 1,2,4,6,8,14 and 21), IFN-beta, IFN-kappa, IFN-omega, and IFN-gamma. Due to unavailability of RNA from samples in our original dataset, these data were derived from skin biopsy samples from an independent cohort of 39 DM patients (see Methods). Importantly, these samples showed similar gene expression patterns to our original dataset (Fig. 4A and S5). We found that expression levels of all IFN transcripts, with the exception of IFN-α4, were elevated in DM skin compared to healthy controls, although there was considerable variation between patients (Fig. 6A). Noting this variation, we next wished to clarify if any of the IFN transcripts were coordinately regulated by performing average linkage hierarchical clustering of the QRT-PCR data for the IFN transcripts across the 39 samples. The IFN-alpha transcripts (with the possible exception of IFN-a21 and IFN-a4) as well as the IFN-omega transcripts were coordinately regulated (Fig. 6B). IFN-beta and IFN-gamma were more closely correlated with one another than any of the other transcripts, while IFN-kappa had the most distinct pattern of expression across the samples.

Our previous data suggested that the IFN signature in DM skin was more similar to the pattern seen with type I rather than type II IFN (Fig. 5). We reasoned that another independent method of identifying the dominant IFN subtype(s) generating the *in vivo* IFN signature in DM skin was to perform a correlation analysis between the level of each particular IFN family transcript and the IFN score for all of the available samples. Strikingly, IFN-beta transcript level was very highly correlated with IFN signature strength in the skin (Spearman r = 0.82; p<0.0001) (Fig. 6C). Interestingly, IFN-gamma also demonstrated a high correlation with IFN signature score (Spearman r = 0.72; p<0.0001), while none of the IFN-alpha subtypes (as well as IFN-omega) were significantly correlated with the strength of the IFN signature (Fig. 6C; data not shown).

## Discussion

This is the first study of global gene expression in the skin of DM patients. We demonstrate an expression signature (the “DM module”) in the skin of 16 DM patients relative to skin from 10 normal, unaffected individuals using whole genome HEEBO oligonucleotide arrays. This DM signature was validated in an independent set of DM skin samples on a different microarray platform (Affymetrix). In addition, examination of this signature across multiple different inflammatory skin diseases demonstrated that this signature is unique to DM and lupus, suggesting that cutaneous DM and cutaneous lupus have a similar underlying pathogenetic mechanism. This signature was characterized by up-regulation of genes involved in antigen processing, complement activation, MHC I/II receptor activity, T cell function, chemokine activity, antiviral and immune responses and epidermal differentiation, and down-regulation of genes involved in fatty acid metabolism, lipid biosynthesis and metabolism, mitochondrial function, and ribosomal function.

One might argue that these gene expression changes are simply a function of increased immune cell infiltration in DM skin. Thus, we performed a study across multiple inflammatory skin diseases to address the specificity of the altered transcriptional modules seen in DM skin. This cross-disease comparison suggests that, while several gene expression patterns are shared more commonly across inflammatory skin diseases, many of the gene expression patterns are not found uniformly across inflammatory skin diseases. For example, upregulation of genes typically associated with epidermal activation was seen in all skin diseases except scleroderma. In addition, we observed downregulation of a large number of genes involved in lipid metabolism across most of the skin diseases we studied. The only disease in which this was not apparent was HSV-2, and this may actually reflect the fact that this dataset used non-lesional skin from patients (rather than skin from healthy volunteers) as a control—downregulation of lipid genes has been seen in non-lesional skin of psoriasis patients [27] and we have also seen this in the uninvolved skin from DM patients (data not shown). Downregulation of genes involved in lipid metabolism has been previously reported in psoriasis and atopic dermatitis [22], [27], [29], but our data suggest that it is a more general feature of many inflammatory skin diseases. It is unclear what cell types are being affected to cause these observed changes, although this lipid regulation could indicate the presence of a defective epidermal barrier, as suggested previously [22]. However, we found other signatures, notably the interferon signature as well as others, that appear to be more characteristic of only selected inflammatory skin diseases, despite the fact that many conditions lacking these signatures (e.g. atopic dermatitis, acne vulgaris) are known to be characterized by inflammatory infiltrates in the skin. Of course, it is still possible that the transcriptional changes seen in DM skin are a direct result of a unique type(s) of infiltrating cell. For the interferon signature, one possible cell type would be the plasmacytoid dendritic cell, and experiments to correlate the strength of various transcriptional responses with infiltrating cell types would be important to perform in the future.

Our data show conclusively that a genuine IFN signature is seen in DM skin, similar to that found in the blood and muscle of DM patients [9], [10], [30], [31]. It is important to look at a complete set of IFN-inducible genes and not simply one gene product, as this can lead to erroneous conclusions of an IFN signature being present. For example, focusing only at MxA, a protein classically characterized as type I IFN-specific, would have led to the conclusion that both atopic dermatitis datasets showed an average of 2.9-fold overexpression relatively to healthy controls (data not shown); this could be misconstrued as evidence of type I IFN activation in the skin of atopic dermatitis, which is clearly not the case. Thus, our results confirm and extend previous reports that demonstrated upregulation of a few selected, IFN-inducible proteins (MxA, CXCL9, CXCL10) in DM skin [15], [16]. This signature was found in all patients in which active skin disease was biopsied, but was found at low or absent levels in biopsies from patients with inactive skin disease, despite the presence of active myositis in the latter group. Thus, the IFN signature in the skin might serve as a useful marker for cutaneous disease activity in DM.

This study also provides information regarding the specificity of the IFN signature across many inflammatory skin diseases. We found that DM, lupus, and HSV-2 skin, and to a lesser degree, psoriasis, had moderate-to-robust IFN signatures, whereas atopic dermatitis, contact dermatitis, systemic sclerosis, and acne vulgaris had relatively weak or absent IFN signatures. Psoriasis was characterized by induction of a large subset of the IFN “core” genes whose expression pattern appeared to correlate with a type I IFN response, in agreement with prior observations [32]. The subtle differences between the IFN signatures of DM and lupus compared with psoriasis may be due to a different source of IFN in the two diseases, different population of responding cells, or modulating cytokines. It is curious that psoriasis appears to have an IFN signature, as all other skin diseases that we found to have this signature are characterized by a predominance of keratinocyte injury and/or death. Indeed, it has been suggested that the IFN signature is characteristic of the lichenoid skin reaction [33], but, given the fact that keratinocyte death is not associated with psoriasis, it is clear that the significance of an IFN signature in skin disease may be more complicated. However, it should be noted that the IFN score was much lower in psoriasis skin than in the other lichenoid disorders, and thus may not reach a threshold required to initiate pathways involved in keratinocyte death.

Our data have highlighted the difficulty in assigning an IFN signature as being “type I” or “type II”, especially in tissue that contains multiple cell types and cannot be easily modeled *in vitro*. Although a type I IFN signature has been suggested to be present in multiple inflammatory skin diseases, this has yet to be shown conclusively [32], [33], [34]. Many genes thought to be specifically induced by type I IFN can also be induced by type II IFN, and this relationship is highly dependent on responding cell type and IFN dose. We have employed two methodologies to address this question in DM skin. First, we used various methods of comparing patterns of IFN-inducible gene expression with those obtained from various *in vitro* stimulations using recombinant type I or type II IFN. Although these comparisons suggest that DM skin is more characteristic of Type I IFN rather than type II IFN activity (see Fig. 5), it is difficult to draw firm conclusions from these data. One weakness of this approach is that it is difficult to compare patterns of gene expression obtained under in vitro conditions in cell lines and that which occurs in a dynamic tissue with multiple responding cell types and unknown concentrations of various IFN proteins. Our data certainly could be consistent with activation of either the type I or II IFN pathways in DM skin.

We also performed a correlation analysis between specific IFN transcript levels and a composite measurement of IFN-activity, namely the IFN gene score. We found that IFN-beta and IFN-gamma levels most closely correlate with activation of IFN-induced genes (see Fig. 6C), also suggesting that either type I or II IFNs could be driving the IFN response. Although we did not find a correlation between the IFN-alpha (and other type I) transcripts we measured, this does not rule out a role for these interferons in DM skin disease. It is possible that a threshold level of IFN-alpha is required for the IFN response, which may then be modulated by varying levels of other interferons. The involvement of specific interferons in disease activity will only be conclusively answered by treating patients with specific IFN inhibitors. An alternative interpretation of these data is that IFN-betaand IFN-gamma) are regulated in the same manner as the other IFN-induced transcripts in DM skin, although this is unlikely given the striking difference in abundance of IFN-beta mRNA versus the other IFN-induced transcripts in DM skin (data not shown). One weakness of this approach is that it assumes that IFN transcript level faithfully reflects the level of active IFN protein in the skin biopsies—however, this assumption would only serve to weaken any positive correlation, and thus the high correlation coefficients we observed are likely of significance.

The fact that many biopsies have high levels of other type I IFN transcripts (which share the same receptor as IFN-beta) in the absence of an IFN signature (Fig. 6C) suggests that other considerations are critical for the strong induction of IFN-induced genes seen in the skin of DM patients—these may include translation of IFN transcripts, localization of IFN production and/or presence of a responder cell population that is sensitive signaling via the IFN receptor. Thus, our data do not rule out an essential role for other type I or II IFNs in the pathogenesis of DM skin disease. They also point out the important fact that elevation of a particular IFN transcript does not provide proof of its activity *in vivo*.

One limitation of our study is that our data are derived from whole skin biopsies, and thus it is unclear what cell(s) constitute the source of IFN, as well as what cell(s) are responsible for producing such high levels of IFN-induced mRNA transcripts. Previous data suggest that keratinocytes as well as inflammatory cells are included in the latter “responding” population in DM skin [16]. However, it is clear that the pattern of expression of IFN-inducible proteins depends on the protein examined [15], [16], [33]. In addition, it will be important to detect the source of different IFN transcripts and proteins in DM skin, which can be technically demanding. Without detailed understanding of these issues, the role of IFN in DM skin disease will remain unclear.

Another potential weakness of our study is that many of our analyses compared data generated by different investigators, using different methodologies, across different microarray platforms. We attempted to mitigate this problem by only using datasets in which a control group was included, so that all data could be expressed relative to healthy patient skin. In addition, where possible, we used identical methodologies for normalizing array data. Despite this, we realize that this can always be a concern when analyzing microarray data, and thus we were more interested in the patterns of gene expression rather than absolute levels of selected genes. We believe our methodology had internal validation in that, for a given disease, independent patient samples run by independent investigators across disparate microarray platforms showed astonishingly similar patterns of gene expression over the gene modules that we were interested in studying (see Figure 3).

Our study defines patterns of gene expression that readily distinguish normal from DM skin. Many of these genes may prove to be useful as specific molecular markers that correlate with disease progression and prognosis. Our analysis provides further insight into the underlying pathogenesis of DM, including characterization of a specific IFN response in DM that may provide more effective targets for therapy.

## Methods

### Ethics Statement

The Stanford Panel on Medical Human Subjects approved human subjects involvement in this research project (last renewal on 04/18/2011). All subjects entered in this study signed an Informed Consent document before donating tissue.

### Patients

All patients were seen in the adult Stanford Dermatology Clinics between August 2004 and March 2006 and agreed to sign an Informed Consent before participating in the study. All patients were diagnosed with DM based upon diagnostic cutaneous findings suggested previously [35] and all patients had a skin biopsy consistent with DM. Exclusion criteria included: (1) age less than 18 years old, and/or (2) evidence of medication-induced DM or mixed-connective tissue disease or other overlap syndromes. There were no restrictions on concomitant medications. We analyzed skin biopsies from 23 DM patients (16 active and 7 inactive) and 10 healthy controls (Table S1). All biopsies were taken from an area that was considered to be representative of active disease. In patients considered to have inactive skin disease (but active myositis), biopsies were taken from areas of telangiectasia and/or pigmentary alteration consistent with prior active DM skin disease. Because skin disease localization in DM patients can be variable, many of these biopsies are from different anatomic sites, although the majority of biopsies were from the arm or neck/upper back (Table S1). 23 DM patients provided a total of 25 biopsies, with 9 technical replicates for a total of 34 samples. 10 healthy controls provided a total of 15 biopsies, with 3 technical replicates, for a total of 18 samples. The DM patients were significantly older than the healthy control patients (mean age 53.1 vs 38.2, p = .019) and had a mean disease duration of 6.0 years. Four of the patients (17%) had cancer-associated DM. 8 of the patients (35%) had skin predominant disease–5 of them with a past history of myositis, and 3 never experiencing myositis (amyopathic). We also included skin biopsies from 4 patients with cutaneous lupus erythematosus (2 with acute cutaneous lupus and 2 with discoid lupus lesions).

### Skin biopsy Processing and Microarray Analysis

Poly-adenylated RNA was extracted from snap-frozen skin biopsy specimens (DM and lupus) using a two step method. First, total RNA was extracted with phenol-guanidine thiocyaniate extraction (RNAbee, Tel-Test, Friendswood TX). Next, poly-adenylated RNA was purified using RNeasy Kit (Quiagen, Chatsworth, CA) according to the manufacturer's instructions. Sample RNA and reference mRNA (Stratagene) were amplified using an Amino Allyl MessageAmp II aRNA kit (Ambion, Austin, TX). Amplified skin RNA (labeled with Cy5) and amplified Human Universal Reference RNA (labeled with Cy3) (Stratagene, La Jolla, CA) were competitively hybridized to HEEBO (human exon evidence-based oligonucleotide) microarrays as described (http://www.microarray.org/sfgf/heebo.do).

Array data were filtered based on fluorescent hybridization signal ≥1.5-fold over local background in either the Cy5 or the Cy3 channel and if the data were technically adequate in ≥80% of experiments. Array data underwent LOWESS normalization, and the data were displayed as a log_2_ ratio of LOWESS-normalized Cy5/Cy3 ratio (span parameter = 0.4). These array data are publicly available at Gene Expression Omnibus (GEO), accession number GSE32245.

For experiments assessing the platform independence of our DM array findings (Fig. 3) and for quantifying IFN expression in DM skin (Fig. 6), several independent DM (n = 39) and healthy control (n = 18) biopsies were obtained in the exact manner as described above. These biopsies were processed and hybridized to Affymetrix Human Genome U133 Plus 2.0 GeneChip arrays as described [32]. These array data were normalized using Robust Multichip Averaging (RMA) using Genespring (www.agilent.com/chem/genespring).

For all datasets, after filtering and normalization, expression data of each sample for all probes mapping to the same UnigeneID were averaged in linear space before any analysis was performed. Genes which showed significantly different expression between DM and controls were identified using Significance Analysis of Microarrays, with a false discovery rate (FDR)<0.043 and a minimum average expression of 2-fold greater or less than controls.

Average linkage hierarchical clustering was performed using GeneCluster ver3.0 software (http://bonsai.ims.u-tokyo.ac.jp/~mdehoon/software/cluster/software.htm). Clustered trees and gene expression heat maps were viewed using Java TreeView Software (http://jtreeview.sourceforge.net/).

### Module Maps

Gene module map was done as described with the program Genomica (http://genomica.weizmann.ac.il/) using gene sets derived from Gene Ontology and KEGG Pathway annotations and the gene expression profiles of skin from patients with dermatomyositis and normal controls.

Briefly, given this collection of precompiled gene sets and this compendium of expression arrays, the method identifies the gene sets that are induced or repressed significantly in the set of arrays (p≤0.05, hypergeometric distribution).

### Comparisons with publicly available microarray data

Publicly available gene expression data was obtained for psoriasis (GSE13355, GSE14905), atopic dermatitis (GSE5667, GSE6012), acne vulgaris (GSE6475), allergic contact dermatitis (GSE6281), HSV-2 infection (GSE18527), systemic sclerosis and morphea (GSE9285), DM muscle (GSE1551, GSE5370), DM blood (courtesy S. Greenberg, Brigham and Women's Hospital), and systemic lupus (GSE8650). The following array platforms were used for these studies: Affymetrix U133A (GSE5667, GSE6012, GSE1551, GSE5370, GSE8650) Affymetrix U133A 2.0 (GSE6475, 5667); Affymetrix U133 plus 2.0 (GSE6281, GSE13355, GSE14905); Agilent Whole Human Genome Oligo Microarray G4112A (GSE9285); Illumina humanref-8 v. 2.0 (GSE18527). Only scleroderma samples that classified as “inflammatory subgroup” were used for comparisons [36], given that we wished to enrich these data for samples that might express an IFN signature. For Affymetrix data, all array data was normalized using Robust Multichip Averaging (RMA) using Genespring (www.agilent.com/chem/genespring).

In order to compare data from different experiments, all expression data were first mapped to a common EntrezGene number. A set of 7783 unique EntrezGene IDs that were common to all platforms was used in these comparisons (“comparison matrix”). Probes that matched to multiple genes were not used. For instances in which multiple probes mapped to the same EntrezGene, these data were averaged by calculating the mean value in linear space to give a single value for each EntrezGene ID. Next, data for each gene in a given disease sample were normalized to the mean expression value of all control samples for that gene. Control samples were always skin biopsies from healthy donors, with the exception of the data from the HSV-2 patients (GSE18527), as the only control data available were derived from patient skin from uninvolved sites. Thus, each experiment was normalized to its own internal control samples, in order to facilitate comparison across experiment sets.

### RT PCR analysis of individual transcripts

The expression level of several microarray target genes was assessed in patient tissue using Taqman quantitative one-step RT-PCR (Applied Biosystems, Foster City, California, United States). Fifty nanograms of total RNA was used in each assay. Assay on demand primers for fatty acid desaturase 1 (Assay ID: Hs00203685_m1), IFN-b1 (Assay ID: Hs00277188_s1), 3-hydroxy-3-methylglutaryl-Coenzyme A synthase 1 (Assay ID: Hs00266810_m1), IFN α 2 (Assay ID: Hs02621172_s1), IFN-a1 (Assay ID: Hs00256882_s1), IFN-induced protein with tetratricopeptide repeats 3 (Assay ID: Hs00155468_m1) and IFN-alpha-inducible protein 6 (Assay ID: Hs00242571_m1) were normalized to GAPDH (Assay ID: Hs99999905_m1) levels and relative abundance was calculated using delta-delta threshold analysis. All assays were conducted on a Stratagene MX3000P thermocycler (La Jolla, CA). RT-PCR analysis of IFN mRNA transcripts was performed as described previously [32].

### Mapping the DM module across disease states

Due to differences across array platforms, we first determined that 490 of the 946 genes from the DM module were present across all samples in the “comparison matrix” (defined above). One-way hierarchical clustering was performed (genes only) and the data were viewed as described above, with 5 representative examples from each inflammatory disease used for comparison.

### Method for defining genes comprising core IFN signature

We reasoned that the skin has keratinocytes, fibroblasts, endothelial cells, and leukocytes, among other cells that could potentially respond differently to IFN. Thus we generated our list of IFN-stimulated genes from all publicly available expression data pertaining to in vitro stimulation with either IFN-gamma, -alpha, -beta, or other IFN preparations—these included data derived from the use of keratinocytes, lung epithelial cells, macrophages, endothelial cells, and fibroblasts as responder cells. These specific datasets used had the following GEO identifiers: GSE1132, GSE7216, GSE5542, GSE3920, GSE1925, GSE1740. The time of stimulation and concentration of IFN varied between studies. For each study, pre- and post-stimulation data were placed in log_2_ space and mean-centered for each gene. All data were then combined and evaluated using Statistical Analysis of Microarrays using a two-class analysis (pre vs post-treatment). From this analysis, 749 genes were identified as significantly regulated by IFN. From this set, we selected only genes that were regulated by an average of two-fold or greater, leaving a final set of 161 genes. Of these 161 genes, 117 were contained in the set of 7783 comprised in the “comparison matrix” gene list discussed above, and thus this set was used as the final “core IFN signature” (Table S4).

### Correlation matrices and principal components analysis

Spearman's rank correlations were calculated between individual arrays for various disease/specimen sources and various *in vitro* stimulations with IFN-alpha or IFN-gamma on different responding cell types using the DM module gene set (490 genes) as well as the IFN signature (as described above). The same expression data of the 117 core IFN signature were further utilized to calculate principal components analysis (PCA). Both the correlations and PCA were conducted using the R statistical computing environment (http://r-project.org). The missing values in the data matrix were imputed by the method *svdImpute* (from the R package “pcaMethods”) and the principal components were calculated with the function *prcomp*.

## Supporting Information

Figure S1 **Inactive DM skin samples cluster with healthy controls and not active DM skin samples.** Shown is a hierarchical clustering dendrogram using two-dimensional clustering of gene expression data from active DM skin (gold branches), skin from patients with inactive skin disease (green branches), and healthy controls (light blue branches). The 946 gene “DM module” was used to cluster the skin samples. The red bar to the right of the dendrogram indicates the cluster of IFN-induced genes.
(TIF)

Figure S2 **Detailed view of heat map of expression of DM module across skin samples.** Two-dimensional hierarchical clustering was performed on gene expression data from active DM skin lesions and skin from healthy controls. A set of 946 genes whose average expression significantly differed between DM and healthy controls (the “DM module) was used to group sample expression data and is shown on the thumbnail diagram at the left. Major clusters of genes are shown with the colored bars and labeled with letters A-G. Shown at the panel on the right are enlarged details of expression data for the labeled clusters, with selected groups of genes labeled at the right. Clusters of genes are: A—epidermal activation; B—leukocyte function; C-IFN signature; D-epidermal differentiation; E—immunoglobulin expression; G—lipid metabolism.
(TIF)

Figure S3 **DM skin module map (detailed).** Module map of the Gene Ontology (GO), Kyoto Encyclopedia of Genes and Genomes (KEGG), and selected other Biological Processes differentially expressed among the active DM samples is shown. Each column represents a single microarray (e.g. patient) and each row represents a single biological process. Only modules that were significantly enriched (minimum 2-fold change, p = 0.05) on at least 4 micoarrays are shown. The average expression of the gene hits from each enriched gene set is displayed here. Only gene sets that show significant differences after multiple hypothesis testing were included. Selected GO or KEGG biological processes are shown. The entire figure with all biological processes can be viewed in Supplementary Figure S3.
(TIF)

Figure S4 **Spearman's rank coefficient matrix using the DM module across different inflammatory disease tissues.** Correlation correlation similarity matrix for 16 cohorts of disease/specimen source groups with 4–5 replicates per each cohort using the 490 genes in the DM module. DM (HEEBO) and DM (Affy) represent data from independent DM skin biopsies run on either HEEBO or Affymetrix arrays, respectively. The remaining datasets were obtained from publicly available GEO omnibus data (see Methods). All data are derived from skin biopsies with the exception of the three diseases on the rightmost region of the x-axis, as indicated. The color legend at the bottom indicates the range of Spearman p values, with general degrees of correlation indicated at each threshold (e.g. low, no (no correlation), mod (moderate), high, or identity). This figure represents the individual array comparisons from the data presented in Figure 3.
(TIF)

Figure S5 **Spearman's rank coefficient matrix using 117 IFN inducible genes both** ***in vitro*** **and** ***in vivo*** **across diseases.** Correlation correlation similarity matrix for 16 cohorts of disease/specimen source groups as well as various *in vitro* stimulations with IFN-alpha or IFN-gamma on different responding cell types with 2–7 replicates per each cohort using 117 IFN inducible genes. Similar to the information presented in Figure 4A, the leftmost columns on the x-axis shows the correlation patterns of this IFN signature following various *in vitro* stimulations with IFN-alpha or IFN-gamma on different responding cell types, as indicated. The rightmost columns on the x-axis show the expression patterns of the IFN signature across multiple disease states. The data from the disease states were derived from publicly available data as described in Figure 3 and Methods. The color legend at the bottom indicates the range of Spearman p values, with general degrees of correlation indicated at each threshold (e.g. low, no (no correlation), mod (moderate), high, or identity). This figure represents the individual array comparisons from the data presented in Figure 4A.
(TIF)

Table S1 **Patients in this study.** ^1^Limited to immunomodulatory medications. ^2^Skin disease graded as active or inactive. Active disease considered based on erythema, induration and/or pruritus. Inactive disease based on absence of erythema with pigmentary alteration and/or telangiectasia. ^3^Defined if any of the following were found: proximal muscle weakness, eleveated muscle enzymes (CPK, aldolase), EMG findings consistent with inflammatory myopathy, muscle biopsy consistent with DM. Abbreviations. DM = DM; NT = not tested; Pred = prednisone (daily dose in mg); MTX = methotrexate; AZA = azathioprine; CSA = cyclosporine; MMF = mycophenolate moefitil; HCQ = hydroxychloroquine; Doxy = doxycycline; homo = homogeneous; unk = unknown; Ca = cancer; ILD = interstitial lung disease.
(DOCX)

Table S2 **List of genes significantly dysregulated in DM skin.** Genes significantly dysregulated in DM skin (compared to skin from healthy donors) were determined using SAM analysis as described in Methods. “Average fold change” refers to the ratio of the mean of the average expression in DM samples divided by the mean of the average expression in control (healthy) samples.
(XLS)

Table S3 **Top 25 upregulated genes in DM skin.** The top 25 upregulated genes from Table S2 are ordered (highest to lowest expression values relative to healthy controls). Genes previously identified to be induced by IFN are bolded.
(XLS)

Table S4 **Core IFN gene list.** The 117 genes comprising the “core IFN gene list” are listed (highest to lowest expression values relative to unstimulated cells). The list was derived as described in Methods.
(XLS)
