# Supplementary figures and images for: Interferon and Biologic Signatures in Dermatomyositis Skin: Specificity and Heterogeneity across Diseases
Source: PLoS One. 2012 Jan 3;7(1):e29161. doi: 10.1371/journal.pone.0029161 (PMC3250414; doi:10.1371/journal.pone.0029161)

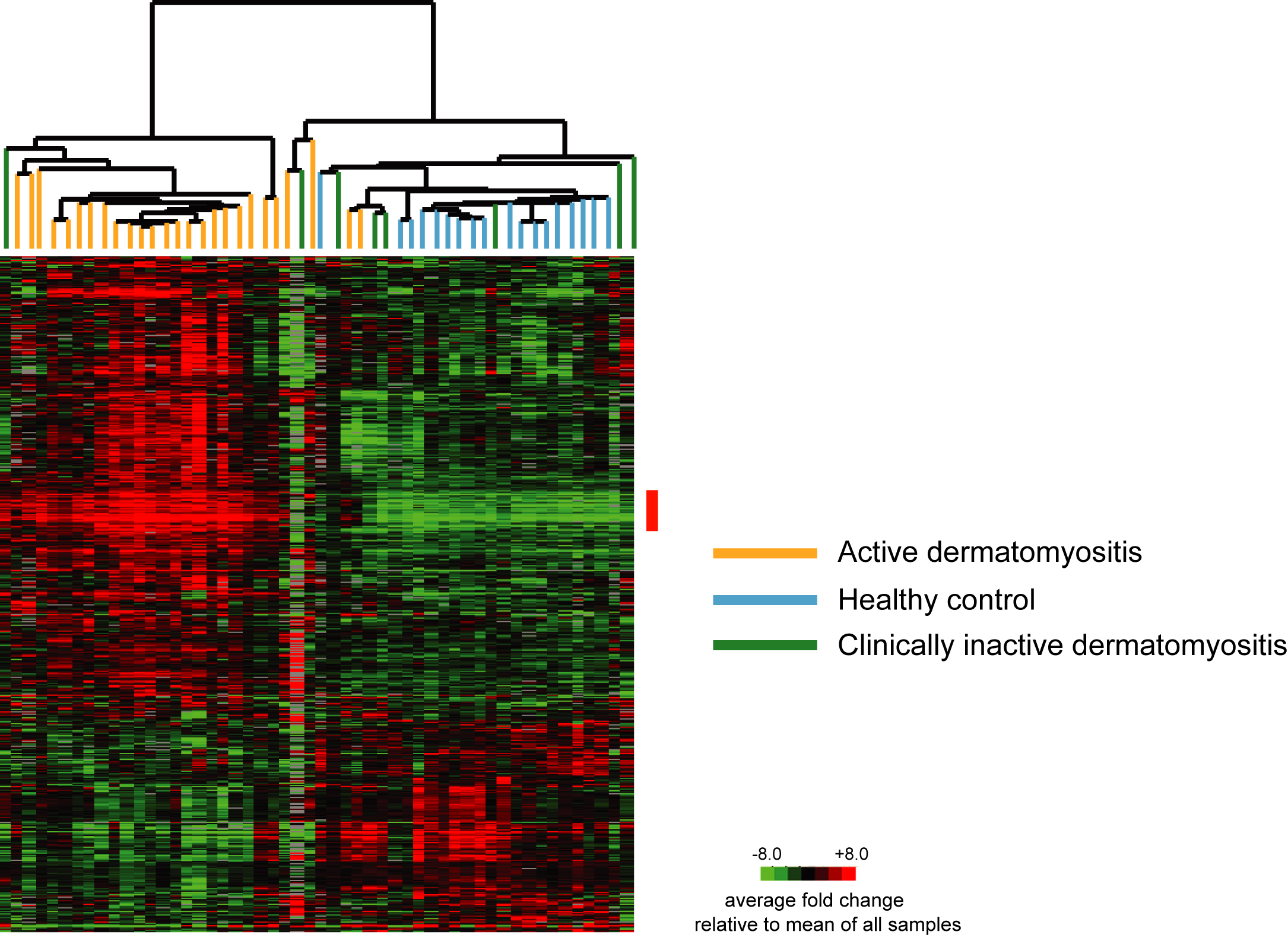

Supplement: Figure S1 — Inactive DM skin samples cluster with healthy controls and not active DM skin samples. Shown is a hierarchical clustering dendrogram using two-dimensional clustering of gene expression data from active DM skin (gold branches), skin from patients with inactive skin disease (green branches), and healthy controls (light blue branches). The 946 gene “DM module” was used to cluster the skin samples. The red bar to the right of the dendrogram indicates the cluster of IFN-induced genes. (TIF) [file pone.0029161.s001.tif]

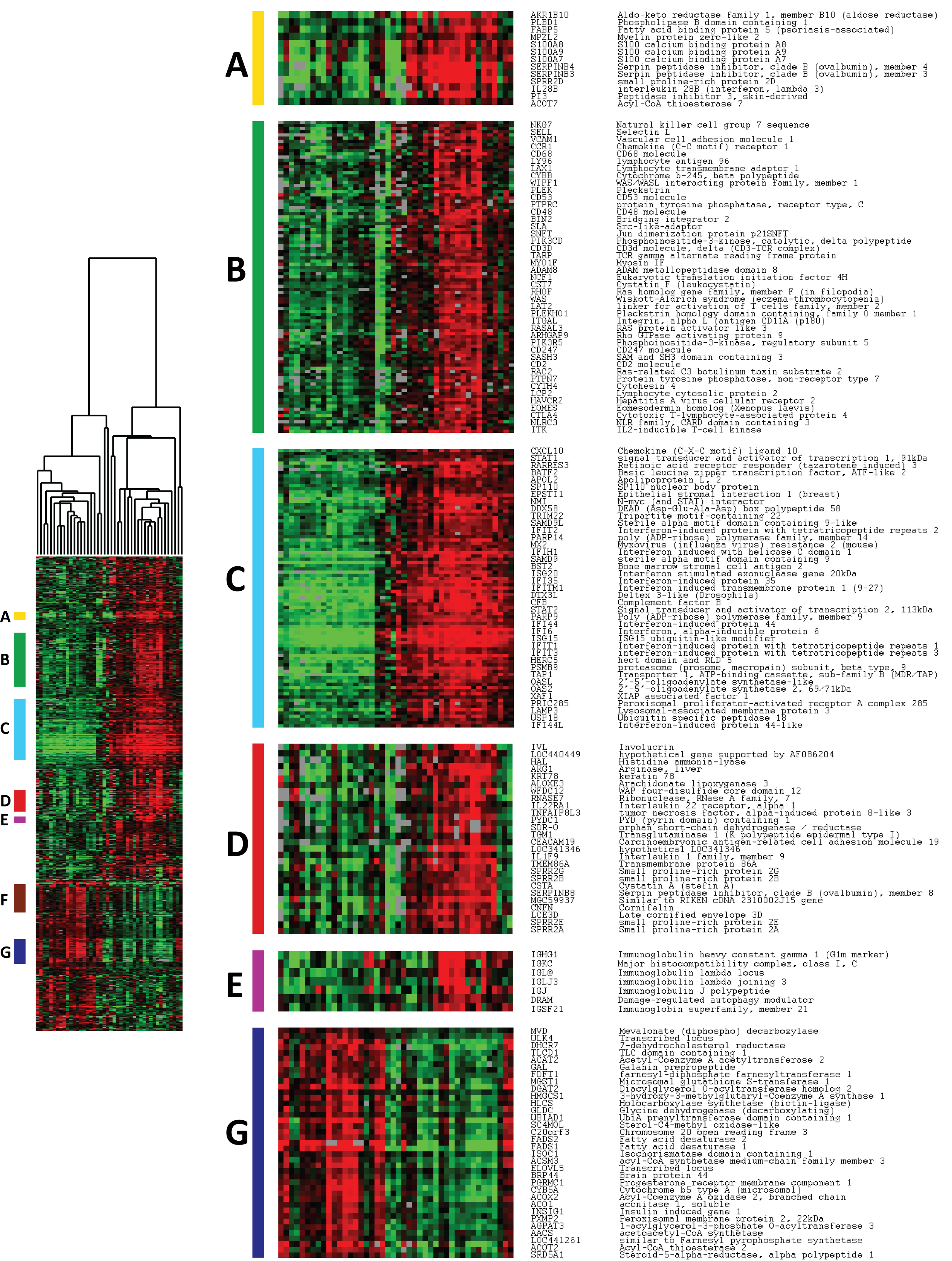

Supplement: Figure S2 — Detailed view of heat map of expression of DM module across skin samples. Two-dimensional hierarchical clustering was performed on gene expression data from active DM skin lesions and skin from healthy controls. A set of 946 genes whose average expression significantly differed between DM and healthy controls (the “DM module) was used to group sample expression data and is shown on the thumbnail diagram at the left. Major clusters of genes are shown with the colored bars and labeled with letters A-G. Shown at the panel on the right are enlarged details of expression data for the labeled clusters, with selected groups of genes labeled at the right. Clusters of genes are: A—epidermal activation; B—leukocyte function; C-IFN signature; D-epidermal differentiation; E—immunoglobulin expression; G—lipid metabolism. (TIF) [file pone.0029161.s002.tif]

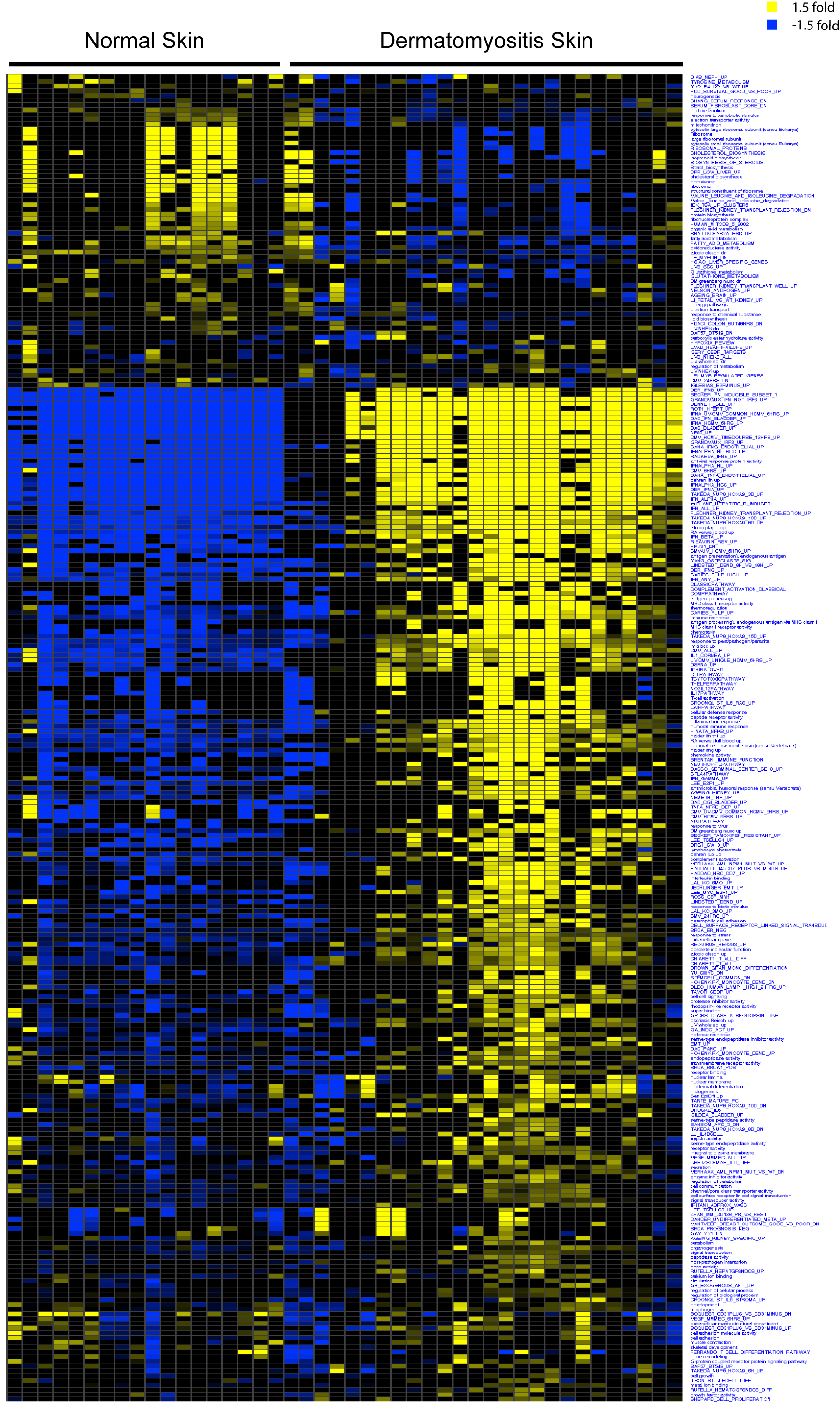

Supplement: Figure S3 — DM skin module map (detailed). Module map of the Gene Ontology (GO), Kyoto Encyclopedia of Genes and Genomes (KEGG), and selected other Biological Processes differentially expressed among the active DM samples is shown. Each column represents a single microarray (e.g. patient) and each row represents a single biological process. Only modules that were significantly enriched (minimum 2-fold change, p = 0.05) on at least 4 micoarrays are shown. The average expression of the gene hits from each enriched gene set is displayed here. Only gene sets that show significant differences after multiple hypothesis testing were included. Selected GO or KEGG biological processes are shown. The entire figure with all biological processes can be viewed in Supplementary Figure S3. (TIF) [file pone.0029161.s003.tif]

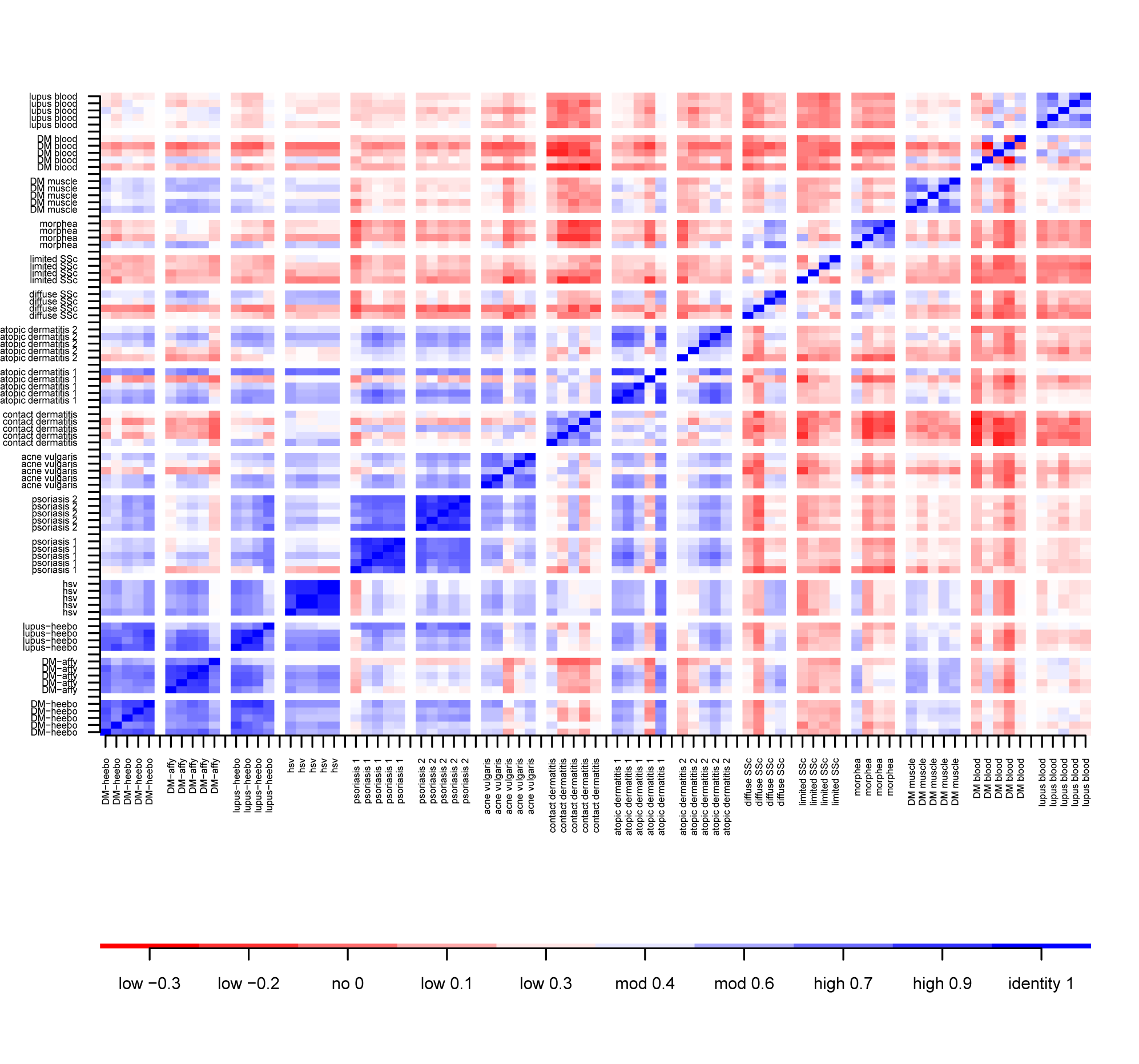

Supplement: Figure S4 — Spearman's rank coefficient matrix using the DM module across different inflammatory disease tissues. Correlation correlation similarity matrix for 16 cohorts of disease/specimen source groups with 4–5 replicates per each cohort using the 490 genes in the DM module. DM (HEEBO) and DM (Affy) represent data from independent DM skin biopsies run on either HEEBO or Affymetrix arrays, respectively. The remaining datasets were obtained from publicly available GEO omnibus data (see Methods). All data are derived from skin biopsies with the exception of the three diseases on the rightmost region of the x-axis, as indicated. The color legend at the bottom indicates the range of Spearman p values, with general degrees of correlation indicated at each threshold (e.g. low, no (no correlation), mod (moderate), high, or identity). This figure represents the individual array comparisons from the data presented in Figure 3. (TIF) [file pone.0029161.s004.tif]

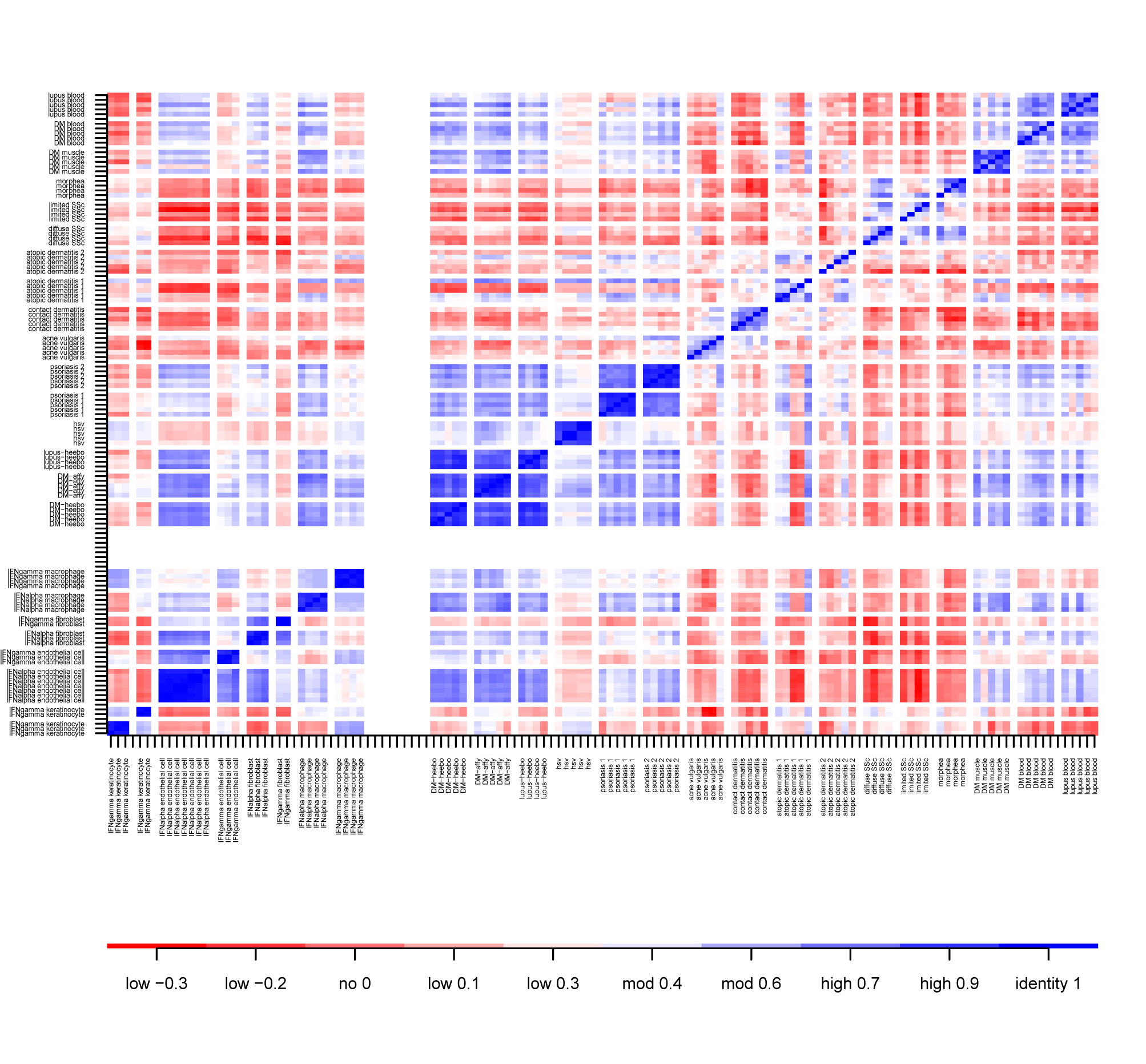

Supplement: Figure S5 — Spearman's rank coefficient matrix using 117 IFN inducible genes both in vitro and in vivo across diseases. Correlation correlation similarity matrix for 16 cohorts of disease/specimen source groups as well as various in vitro stimulations with IFN-alpha or IFN-gamma on different responding cell types with 2–7 replicates per each cohort using 117 IFN inducible genes. Similar to the information presented in Figure 4A, the leftmost columns on the x-axis shows the correlation patterns of this IFN signature following various in vitro stimulations with IFN-alpha or IFN-gamma on different responding cell types, as indicated. The rightmost columns on the x-axis show the expression patterns of the IFN signature across multiple disease states. The data from the disease states were derived from publicly available data as described in Figure 3 and Methods. The color legend at the bottom indicates the range of Spearman p values, with general degrees of correlation indicated at each threshold (e.g. low, no (no correlation), mod (moderate), high, or identity). This figure represents the individual array comparisons from the data presented in Figure 4A. (TIF) [file pone.0029161.s005.tif]
