# Supplementary material for: Interferon and Biologic Signatures in Dermatomyositis Skin: Specificity and Heterogeneity across Diseases
Source: PLoS One. 2012 Jan 3;7(1):e29161. doi: 10.1371/journal.pone.0029161 (PMC3250414; doi:10.1371/journal.pone.0029161)
Supplement: Table S1 — Patients in this study. 1Limited to immunomodulatory medications. 2Skin disease graded as active or inactive. Active disease considered based on erythema, induration and/or pruritus. Inactive disease based on absence of erythema with pigmentary alteration and/or telangiectasia. 3Defined if any of the following were found: proximal muscle weakness, eleveated muscle enzymes (CPK, aldolase), EMG findings consistent with inflammatory myopathy, muscle biopsy consistent with DM. Abbreviations. DM = DM; NT = not tested; Pred = prednisone (daily dose in mg); MTX = methotrexate; AZA = azathioprine; CSA = cyclosporine; MMF = mycophenolate moefitil; HCQ = hydroxychloroquine; Doxy = doxycycline; homo = homogeneous; unk = unknown; Ca = cancer; ILD = interstitial lung disease. (DOCX) [file pone.0029161.s006.docx]

Table S1. Patients in this study.

| Patient | Age/  Gender | Diagnosis | Disease  duration | Current Medications^1^ | | Skin disease^2^ | Biopsy site | Myositis^3^ | ANA | Comorbid  conditions | |
| --- | --- | --- | --- | --- | --- | --- | --- | --- | --- | --- | --- |
| 25 | 23/F | Healthy | -- | -- | | -- | forearm | -- | NT | | -- |
| 122 | 53/F | Healthy | -- | -- | | -- | cheek | -- | NT | | -- |
| 124 | 35/F | Healthy | -- | -- | | -- | lower back | -- | NT | | -- |
| 130 | 53/M | Healthy | -- | -- | | -- | a:forearm  b:buttock | -- | NT | | -- |
| 133a | 28/F | Healthy | -- | -- | | -- | arm | -- | NT | | -- |
| 134a | 45/M | Healthy | -- | -- | | -- | posterior neck | -- | NT | | -- |
| 135 | 70/M | Healthy | -- | -- | | -- | cheek | -- | NT | | -- |
| 137 | 28/M | Healthy | -- | -- | | -- | a:arm  b:posterior neck  c:buttock | -- | NT | | -- |
| 141 | 22/M | Healthy | -- | -- | | -- | a:arm  b:posterior neck | -- | NT | | -- |
| 142 | 25/F | Healthy | -- | -- | | -- | a:buttock  b:posterior neck | -- | NT | | -- |
| 7 | 65/F | DM | 3.3 y | Pred10, MMF, HCQ | | active | posterior neck | past | 1:80 (speckled) | | ILD |
| 10b | 61/F | DM | 18.5 y | Pred5, MTX, CSA, HCQ, AZA | | active | posterior neck | past | negative | | -- |
| 11 | 47/F | DM | 3 y | Pred 10 | | active | a:posterior neck  b:arm | past | negative | | -- |
| 20 | 65/M | DM | 1.7 y | HCQ | | active | finger  (gottron) | present | negative | | -- |
| 33 | 59/F | DM | 6 mo | Pred 5 | | active | arm | never | 1:80 (nucleolar) | | Ca |
| 35 | 42/F | DM | 2 y | Pred10, MTX | | active | arm | present | 1:1280 (speckled) | | -- |
| 39 | 76/M | DM | 1 y | Pred40 | | active | posterior neck | present | 1:160 | | Ca |
| 59 | 42/M | DM | 15 mo | MMF, Doxy | | active | upper back | present | negative | | -- |
| 60 | 74/F | DM | 7.5 y |  | | active | thigh | past | 1:160 (homo) | | -- |
| 88 | 62/M | DM | 10 mo | Pred60, HCQ | | active | knee | never | negative | | Ca |
| 89 | 73/F | DM | 1 y | MMF | | active | upper back | present | 1:160 (speckled) | | -- |
| 103 | 55/F | DM | 1 y | Pred1, MTX | | active | chest | present | negative | | -- |
| 104 | 71/F | DM | 3 y | Pred5, MTX | | active | forearm | past | 1:1280 (homo) | | -- |
| 110 | 24/F | DM | 9 mo |  | | active | finger  (gottron) | never | 1:160 (speckled) | | -- |
| 121 | 44/M | DM | 3 y | Pred40, AZA | | active | forearm | present | negative | | Ca,ILD |
| 129 | 51/F | DM | 4 y |  | | active | a:posterior neck  b:buttock | present | 1:320 (speckled) | | -- |
| 36 | 64/M | DM | 3 y | Pred80 | | active | dorsal hand | present | unk | | -- |
| 62 | 21/M | DM | 6 y | Pred16, MTX, Doxy | | inactive | chest | present | negative | | -- |
| 65 | 65/M | DM | 2 y | Pred12.5, AZA, HCQ | | inactive | posterior neck | present | negative | | -- |
| 66 | 35/M | DM | 7.5 y | Pred15, AZA | | inactive | chest | present | 1:160 | | -- |
| 77 | 37/M | DM | 14 y |  | | inactive | neck | present | unk | | -- |
| 78 | 42/M | DM | 5 y | Pred25, MTX | | inactive | elbow | present | unk | | -- |
| 79 | 46/M | DM | 11 y | Pred15, AZA | | inactive | neck | present | unk | | -- |
|  | | | | |  |  |  |  |  |  |  |
